# Semi-automated analysis of infarct heterogeneity on DE-MRI using graph cuts

**DOI:** 10.1186/1532-429X-14-S1-T6

**Published:** 2012-02-01

**Authors:** YingLi Lu, Kim A Connelly, Yuesong Yang, Subodh B Joshi, Graham Wright, Perry E Radau

**Affiliations:** 1 Imaging Research, Sunnybrook Health Sciences Centre, Toronto, ON, Canada; 2 Keenan Research Centre in the Li Ka Shing Knowledge Institute, St. Michael’s Hospital and University of Toronto, Toronto, ON, Canada; 3 Cardiology, Sunnybrook Health Sciences Centre, Toronto, ON, Canada; 4 Medical Biophysics, University of Toronto, Toronto, ON, Canada

## Background

Two popular methods for determining the threshold values for the infarct core and gray zone on delayed enhancement MR images (DE-MRI) have been proposed previously: full width and half maximum (FWHM) and standard deviation (SD) methods [1]. Major limitations of these methods are:1) three manually drawn contours are needed for endocardial, epicardial and remote myocardium boundaries, which is time consuming and suffers from inter-observer and intra-observer variability; 2) the difficulty in reproducible manual delineation of remote myocardium, is an important contributor to variability in results; and 3) the dependence on the remote region statistics is problematic due to the low SNR of this region [2]. The purpose of this research was to develop a novel algorithm for segmentation of infarct core and gray zone from conventional IR-GRE short-axis MR images with highly robust and reproducible results comparable to the FWHM analysis while eliminating the requirement for drawing a remote myocardial region.

## Methods

**Figure 1:**
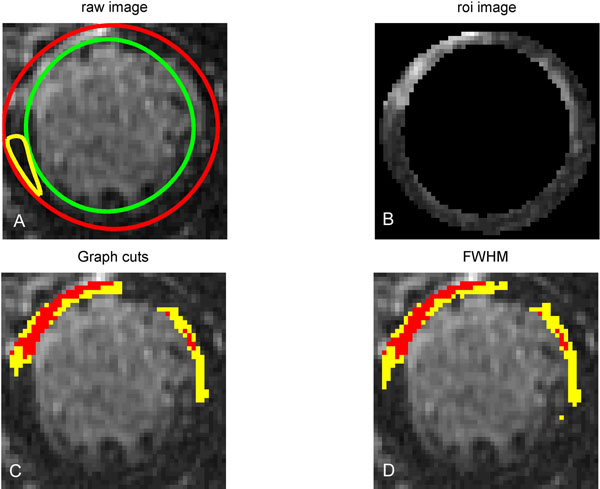
Infarct core and gray zone segmented with graph cuts compared with FWHM methods. A: Automated epicardial (red), endocardial(green) and remote myocardium(yellow). B: ROI Image. C: Infarct core (red) and gray zone(yellow) segmented with graph cuts (without remote myocardium). D: Infarct core (red) and gray zone (yellow) segmented with FWHM.

**Figure 2:**
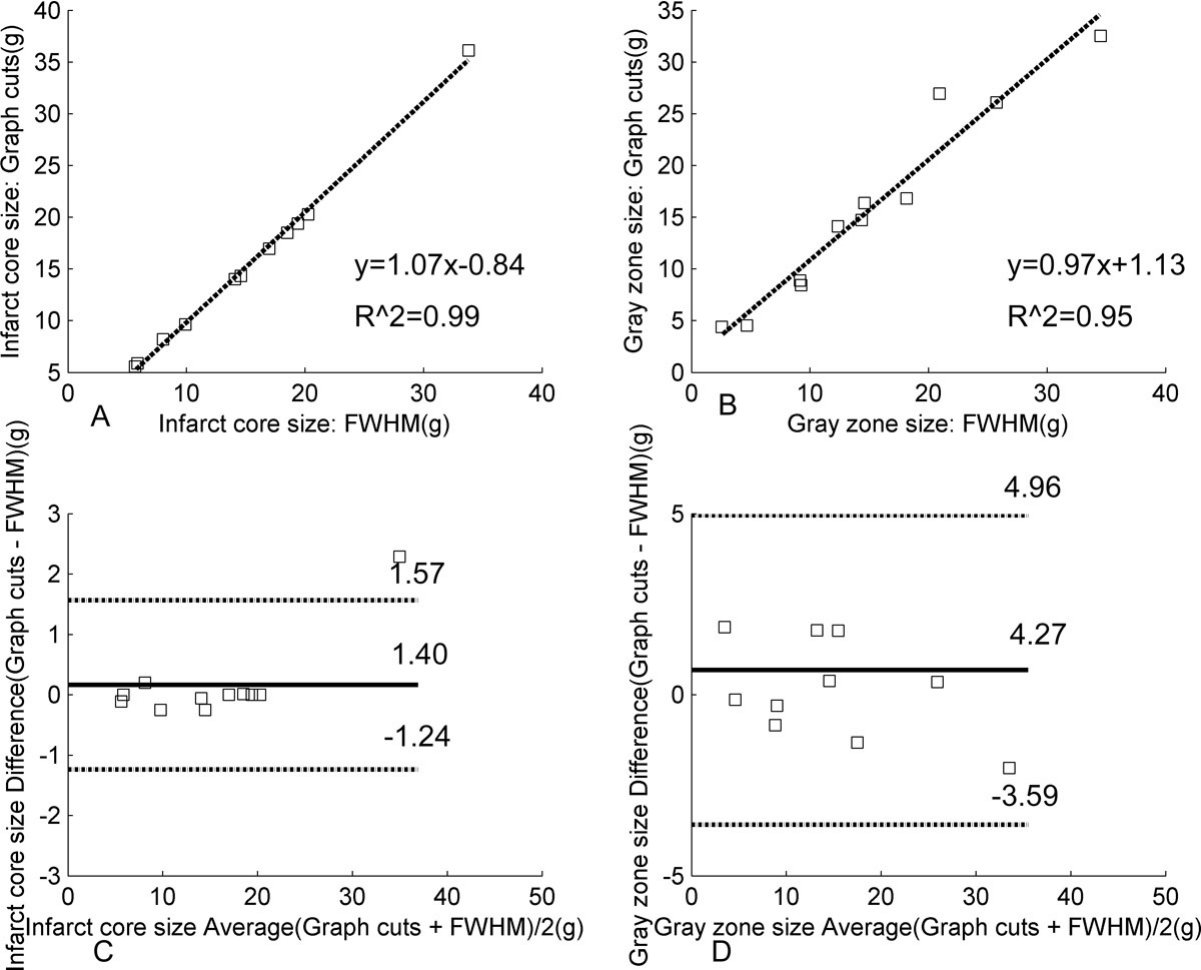
Linear regression between graph cuts and FWHM calculation of infarct mass for infarct core (A) and gray zone (B). Bland-Altman plots comparing graph cuts and FWHM calculations of infarct core (C) and gray zone (D). The horizontal solid line is bias, and dashed lines are the limits of agreement (+/- 1.96 SD).

Eleven male patients (age: 63.5±11.8 yr) with known CAD and evidence of LGE and chronic MI had cardiac IR-GRE MR scans with full left ventricle (LV) coverage (7-13 slices). MR imaging was performed on a 1.5 T scanner (CV/i, GE Healthcare) using an 8-channel cardiac coil. DE-MRI was started 10 min after the injection of 0.2 mmol/kg of Gd-DTPA (Magnevist, Berlex). First, the endocardial and epicardial contours were generated from the corresponding SSFP images automatically [3] with papillary muscles included. (42%(54/136) of the contours need manual adjustment. Next, the graph cuts algorithm [4] was used to segment the infarct: 1) a two-classes Gaussian Mixture Model (GMM) was created for the myocardial ROI, determined by the endocardial and epicardial contours; 2) each pixel was assigned to the most likely Gaussian component; and 3) a graph was built and the graph cut algorithm finds the optimum classification of healthy and infarcted myocardium. Finally, the segmented infarct is separated into infarct core and gray zone using a threshold of half the maximal signal within the segmented infarct (Fig. 1). Linear regression analysis and Bland-Altman plots were used to compare the FWHM method (requiring manual ROIs for LV and remote myocardium) and our method (Fig. 2).

## Results

There were excellent correlations of the infarct size (infarct core 1: R^2 = 0.99; gray zone: R^2 = 0.95) derived from our graph cuts method and the manual FWHM method. The Bland-Altman analysis indicated that there was a small overestimation bias (infarct core: 0.17 g; gray zone: 0.68 g) with limits of agreement of ± 1.40 g (infarct core) and ± 4.27 g (gray zone). This variability is small relative to the reported range of gray zone masses (20 +/- 13 g, N=91 [2]).

## Conclusions

The results for the proposed semi-automated segmentation technique indicate that it will streamline accurate quantification of myocardial infarct on IR-GRE MR infarct images in clinical practice.

## Funding

The authors thank the Canadian Foundation for Innovation (CFI), the Canadian Institutes of Health Research (CIHR),and MaRS Innovation Proof of Principle Program for their support.
